# G protein γ subunit *qPE9-1* is involved in rice adaptation under elevated CO_2_ concentration by regulating leaf photosynthesis

**DOI:** 10.1186/s12284-021-00507-7

**Published:** 2021-07-15

**Authors:** Ke Wang, Feiyun Xu, Wei Yuan, Leyun Sun, Shaoxian Wang, Mehtab Muhammad Aslam, Jianhua Zhang, Weifeng Xu

**Affiliations:** 1 College of Life Sciences, Joint International Research Laboratory of Water and Nutrient in Crop and College of Resources and Environment, Fujian Agriculture and Forestry University, 350002 Fuzhou, China; 2 Department of Biology, Hong Kong Baptist University, Hong Kong, China

**Keywords:** Elevated CO_2_, G protein, *qPE9-1*, Rubisco, Rice

## Abstract

G protein γ subunit *qPE9-1* plays multiple roles in rice growth and development. However, the role of *qPE9-1* in rice exposed to elevated carbon dioxide concentration (eCO_2_) is unknown. Here, we investigated its role in the regulation of rice growth under eCO_2_ conditions using *qPE9-1* overexpression (OE) lines, RNAi lines and corresponding WT rice. Compared to atmospheric carbon dioxide concentration (aCO_2_), relative expression of *qPE9-1* in rice leaf was approximately tenfold higher under eCO_2_. Under eCO_2_, the growth of WT and *qPE9-1-*overexpressing rice was significantly higher than under aCO_2_. Moreover, there was no significant effect of eCO_2_ on the growth of *qPE9-1* RNAi lines. Furthermore, WT and *qPE9-1-*overexpressing rice showed higher net photosynthetic rate and carbohydrate content under eCO_2_ than under aCO_2_. Moreover, the relative expression of some photosynthesis related genes in WT, but not in RNAi3 line, showed significant difference under eCO_2_ in RNA-seq analysis. Compared to WT and RNAi lines, the *rbcL* gene expression and Rubisco content of rice leaves in *qPE9-1-*overexpressors were higher under eCO_2_. Overall, these results suggest that *qPE9-1* is involved in rice adaptation under elevated CO_2_ concentration by regulating leaf photosynthesis via moderating rice photosynthetic light reaction and Rubisco content.

**Supplementary Information:**

The online version contains supplementary material available at 10.1186/s12284-021-00507-7.

## Background

Atmospheric carbon dioxide concentration (aCO_2_) has increased at a rate of 2 ppm/year since 2002. Currently, aCO_2_ has exceeded 400 ppm (Meinshausen et al. 2011) and it is expected to reach 550–700 ppm by 2050. In addition, human population will reach ten billion by 2050 (United Nations 2015), which will lead to overexploitation of natural resources. It will be a big challenge for us to intensify agro-productions to feed this growing population. Thus, a better understanding of growth under elevated carbon dioxide concentration (eCO_2_) leading to increased growth is essential (Kimball 2016), which can help breeders to improve crop germplasm for climate change.

Rice (*Oryza sativa* L.) is a major staple food crop for almost half of the global population (Kurai et al. 2011). Exposure to eCO_2_, rice yield is improved by increasing plant growth, tiller number and leaf area (Kimball 2016; Hasegawa et al. 2013). In addition, the gas exchange and net photosynthetic rate increase under eCO_2_ conditions (Norby et al. 2016). Different studies have been employed to evaluate the effect of eCO_2_ on crops, but the underlying molecular mechanisms and signaling need to be probed (Becklin et al. 2017). CO_2_ enrichment showed great effect on biological processes included protein phosphorylation, protein ubiquitination, oxidation-reduction and plant organ development (Ge et al. 2018). Under eCO_2_, genes involved in CO_2_ fixation showed lower gene expression, but genes involved in ribulose-1,5-bisphospate generation and starch synthesis showed higher gene expression (Fukayama et al. 2009). *ATL31* expression was induced in response to high CO_2_/low N condition in senescence progression (Aoyama et al. 2014). Some genes have been previously studies for their functional involvement in eCO_2_ response in plants. Ribulose-1,5-bisphosphate carboxylase/oxygenase (Rubisco) is a key enzyme for CO_2_ fixing into the Calvin cycle. The RNAi-mediated down-regulation of the small Rubisco subunit (*rbcS)* significantly decreased rice photosynthetic rate and biomass under aCO_2_ conditions, however, under eCO_2_, the *rbcS* RNAi lines showed higher net photosynthetic rate and biomass than WT (Kanno et al. 2017; May et al. 2013) demonstrated that miR156/157 and miR172 were involved in early flowering induction by eCO_2_. In addition, we reported previously that overexpression of *OsPIP1;2* resulted in 15–20 % biomass increase when grown under eCO_2_ (Xu et al. 2019). In a previous study, *CRCT* was reported to play a key role in regulating the expression of CO_2_-responsive genes (Morita et al. 2017). *SCRM2* and *CDKB1* regulated stomatal patterning in response to eCO_2_ (Watson-Lazowski et al. 2016). Lastly, a rice small GTPase, *Rab6a*, encodes a monomeric G protein related to the α-subunit of G proteins and is involved in the regulation of grain yield in response to eCO_2_ (Yang et al. 2020). Overexpression of *OsRab6a* significantly increased rice net photosynthetic rate, biomass and grain yield under eCO_2_ condition.

Heterotrimeric GTP-binding proteins (G proteins) are composed of Gα, Gβ and Gγ subunits, and mediated a variety of growth and developmental processes in plants, including extracellular signal transduction, ion channel regulation, abiotic stresses, cell proliferation, cell wall modification and responses to phytohormones (Li et al. 2012; Jones and Assmann 2004; Klopffleisch et al. 2011; Swain et al. 2017; Yadav et al. 2012; Choudhury et al. 2013; Chakravorty et al. 2011; Trusov et al. 2009; Subramaniam et al. 2016). Rice has one Gα (*RGA1*), one Gβ (*RGB1*), and five Gγ (*RGG1*, *RGG2*, *GS3*, *DEP1/qPE9-1*, and *GGC2*) genes (Sun et al. 2018). *qPE9-1*, which is allelic to *DEP1*, showed functions in panicle (Huang et al. 2009; Zhou et al. 2009; Sun et al. 2014) showed that rice carrying the dominant *dep1-1* allele exhibited nitrogen insensitive vegetative growth. *qPE9-1* also positively regulated starch accumulation and enhanced the accumulation of auxin and cytokinin phytohormones during grain filling stage (Zhang et al. 2019). However, the role of *qPE9-1* in plant growth under elevated CO_2_ concentration (eCO_2_) is unknown.

In the present study, we evaluated the role of *qPE9-1* in plant growth in response to eCO_2_ using overexpression (OE), RNAi lines of *qPE9-1* and wild-type (WT) rice. In addition, net photosynthetic rate, carbohydrate content, and Rubisco content of OE lines, RNAi lines of *qPE9-1* and WT were determined under aCO_2_ and eCO_2_ conditions. We aimed to determine the role of *qPE9-1* in rice under eCO_2_ and its potential application in agriculture.

## Results

### Expression pattern of ***qPE9-1*** in rice plants

**Fig. 1:**
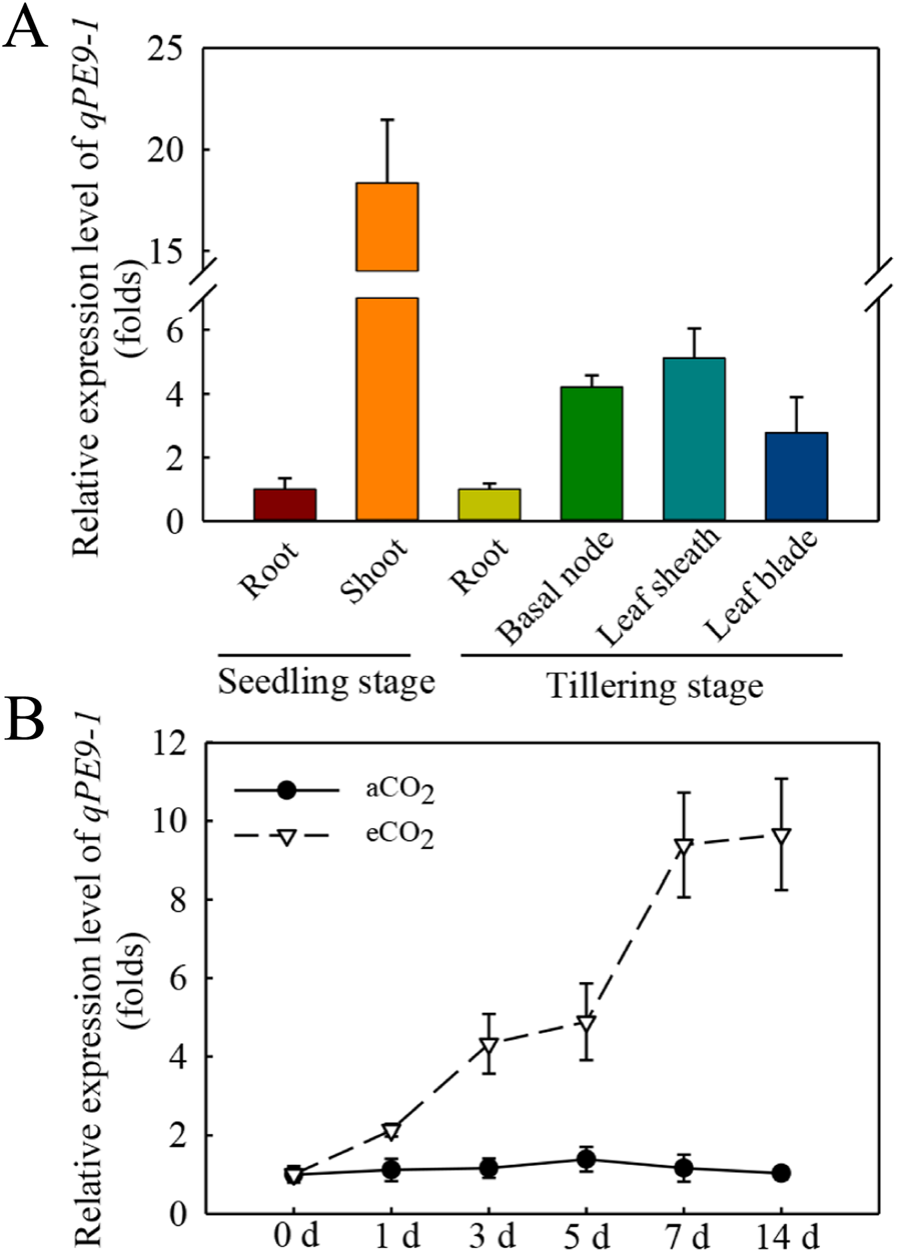
Analysis of *qPE9-1* expression pattern under aCO_2_ or eCO_2_ using RT-qPCR in the rice plants. (**A**) Expression levels of *qPE9-1* in the indicated tissues of WT at seedling and tillering growth stages under aCO_2_. The shoots included leaf blade and leaf sheath at seedling stage. The leaf sheath and leaf blade of newly expanded leaf were taken for the measurement at tillering stage. (**B**) Time course of *qPE9-1* expression at seedling stage after exposed to eCO_2_. WT seedlings were transferred to aCO_2_ and eCO_2_ conditions and then the leaves samples were harvested at the indicated time points for RNA extraction. The value before treatment (0 d) set as 1, and then gene expression is relative to it. *OsActin* was as an internal reference. Values are means ± SD; different letters indicate significant differences (*P* < 0.05)

Tanaka et al. (2016) reported that CCRE1/2/3 *cis*-elements (TGACGT, ACGTCA, and TGACGC) were identified to be CO_2_ responsive elements. In the present study, we found that the promoter sequence of *qPE9-1* had the CCRE3 (TGACGC) element (Table S4). At the seedling stage, the gene expression of *qPE9-1* showed higher expression in the shoot. In addition, the gene expression of *qPE9-1* in leaf sheath and basal node were the highest at the tillering stage (Fig. 1 A). To investigate the physiological and functional relevance of *qPE9-1*, we examined the gene expression of *qPE9-1* under aCO_2_ and eCO_2_ conditions. It was found that, after eCO_2_ treatment, the *qPE9-1* expression increased rapidly at 1 d (2.1-fold). Subsequently, however, the gene expression of *qPE9-1* remained nearly unchanged at 3 d and 5 d and plateaued off at 7 d and 14 d after eCO_2_ treatment (Fig. 1B). Moreover, the relative expression of *qPE9-1* did not significantly increase under aCO_2_ at the indicated time points, suggesting that the relative expression of *qPE9-1* is due to the elevated CO_2_ concentration. Moreover, the expression level of other subunits (*RGA1*, *RGB1*, *RGG1*, *RGG2*, *GS3*, *GGC2*) were not induced by eCO_2_ (Fig. S1).

### Growth response of ***qPE9-1*** knockdown and overexpression lines to eCO_2_

**Fig. 2:**
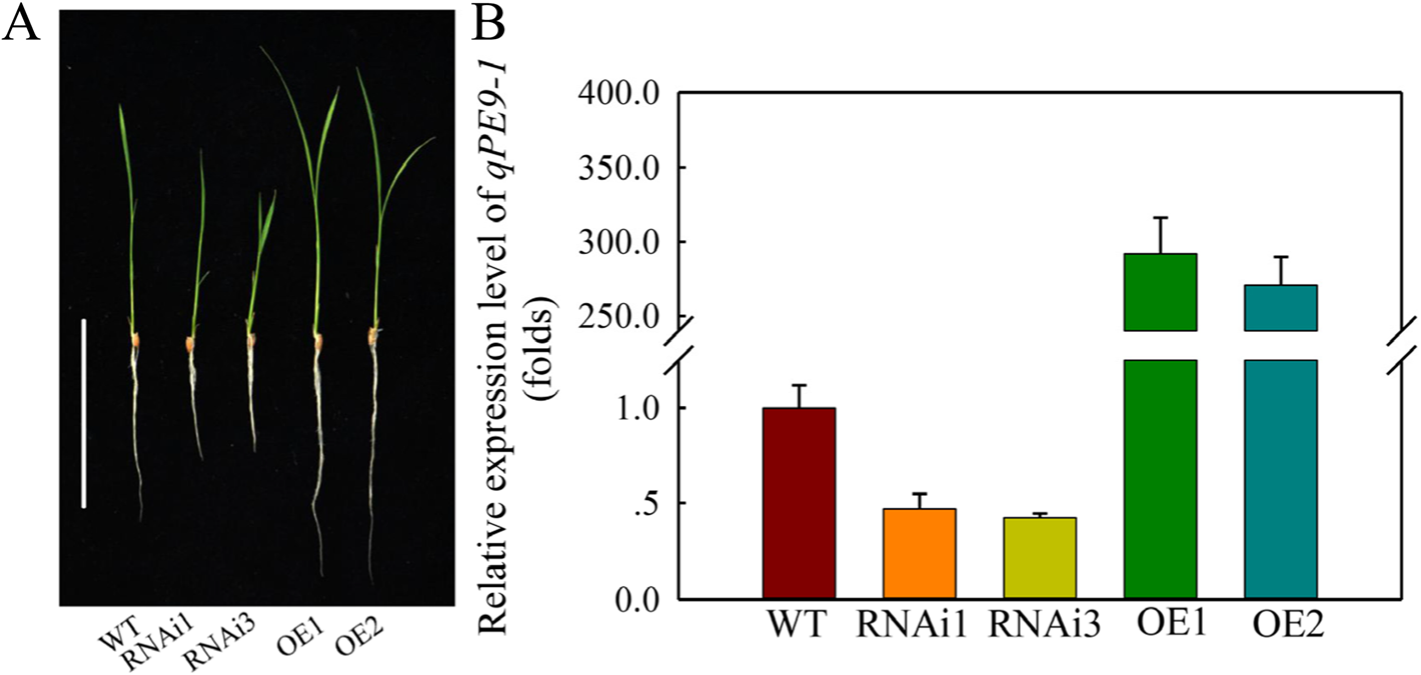
Phenotype and expression level of *qPE9-1* in wild-type rice and transgenic lines. (**A**) Phenotype of 7-d-old transgenic lines (WT, RNAi lines RNAi1 and RNAi3 or overexpressing lines OE1 and OE2 of *qPE9-1*). Scale bar: 10 cm. (**B**) Expression level of *qPE9-1* in the RNAi and OE lines by real-time quantitative PCR with *OsActin* as an internal reference. Values are means ± SD (*n* = 3); different letters indicate significant differences (*P* < 0.05)

To characterize the physiological function of *qPE9-1* in rice, the OE, RNAi lines and WT were used in the study. The relative expression of *qPE9-1* in OE lines was significantly (270–290 fold) higher than in WT (Fig. 2B). In addition, the relative expression of *qPE9-1* was markedly lower in the RNAi lines than in the WT.

**Fig. 3:**
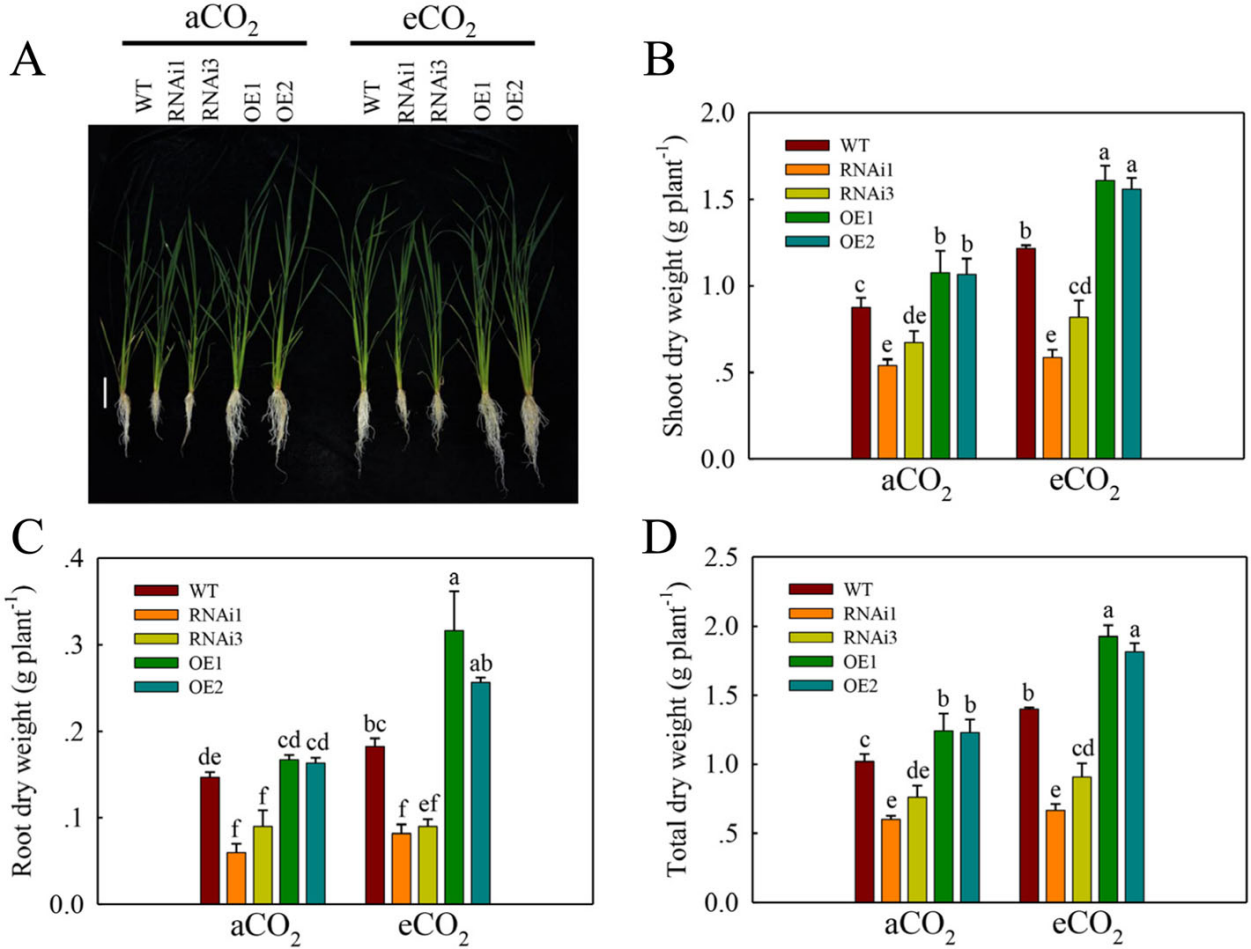
Growth of wild-type rice and transgenic lines under aCO_2_ and eCO_2_. Seedlings were grown under aCO_2_ (400 ppm) or eCO_2_ (800 ppm) in growth chambers for 4 weeks. (**A**) Growth phenotypes of WT, RNAi lines and OE lines of *qPE9-1* under aCO_2_ or eCO_2_ for 4 weeks. Bar: 10 cm. (**B**, **C** and **D**) Shoot dry weight, root dry weight and total dry weight of WT, RNAi lines and OE lines of *qPE9-1* grown under aCO_2_ or eCO_2_ for 4 weeks. Values are means ± SD (*n* = 5); different letters indicate significant differences (*P* < 0.05)

We next examined the growth of the OE lines, RNAi lines of *qPE9-1*and WT under aCO_2_ and eCO_2_ conditions (Fig. 3). The shoot dry weight, root dry weight and total dry weigh of the OE lines and WT were significantly higher under eCO_2_ than under aCO_2_ (Fig. 3). However, the dry weight of RNAi lines showed no significant difference under both CO_2_ conditions. The shoot dry weight, root dry weight and total dry weight of the OE lines were 10–18 %, 17–20 %, and 18–21 % higher, respectively, than those of WT under aCO_2_. Under eCO_2_, the shoot dry weight, root dry weight and total dry weight of the OE lines were 19–28 %, 24–34 %, and 25–43 % higher than those of WT.

### Effect of eCO_2_ on rice gas-exchange parameters

**Fig. 4:**
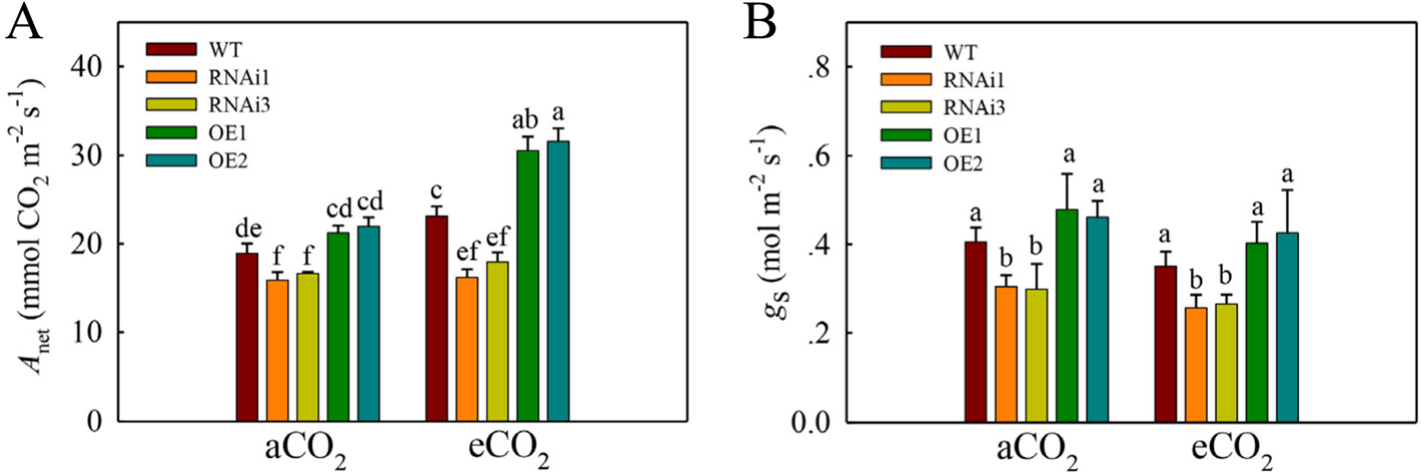
Effects of eCO_2_ on the net rate of CO_2_ assimilation (*A*_net_) and stomatal conductance (*g*_s_) in WT, RNAi lines and OE lines of *qPE9-1*. Rice plants (the OE lines, WT and RNAi lines) were grown under aCO_2_ or eCO_2_ in growth chambers for 4 weeks. (**A**) Net rate of CO_2_ assimilation (*n* = 5). (**B**) stomatal conductance (*g*_s_) (*n* = 5). All values are means ± SD; different letters indicate significant differences (*P* < 0.05)

When exposed to eCO_2_, plant growth changes partially related to the immediate effect of eCO_2_ on photosynthesis and stomatal conductance (Gamage et al. 2018). Therefore, the gas exchange parameters were investigated in OE lines, RNAi lines of *qPE9-1* and WT (Fig. 4). Compared to aCO_2_, the net rate of CO_2_ assimilation (*A*_net_) in WT and OE lines was significantly increased in the presence of eCO_2_, respectively (Fig. 4). However, the *A*_net_ of RNAi lines was not different under aCO_2_ and eCO_2_ conditions. The *A*_net_ in the OE lines was 12–16 % higher, respectively, than WT under aCO_2_ (Fig. 4 A). In addition, under eCO_2_, the *A*_net_ in the OE lines was 23–27 % higher than in WT (Fig. 4 A). Moreover, the *A*_net_ of WT under eCO_2_ was 22 % higher than under aCO_2_. The *A*_net_ of OE lines under eCO_2_ was 43 % higher than those under aCO_2_ (Fig. 4 A). In contrast to *A*_net_, the stomatal conductance (*g*_s_) of WT and transgenic lines was reduced under eCO_2_ conditions, but there were significant differences among WT, RNAi, and OE lines under aCO_2_ and eCO_2_ conditions (Fig. 4B). In addition, *A*_net_/*g*_s_ of OE lines was significantly higher than of WT and RNAi lines under eCO_2_ (Fig. S2).

### Effect of eCO_2_ on carbohydrate content

**Fig. 5:**
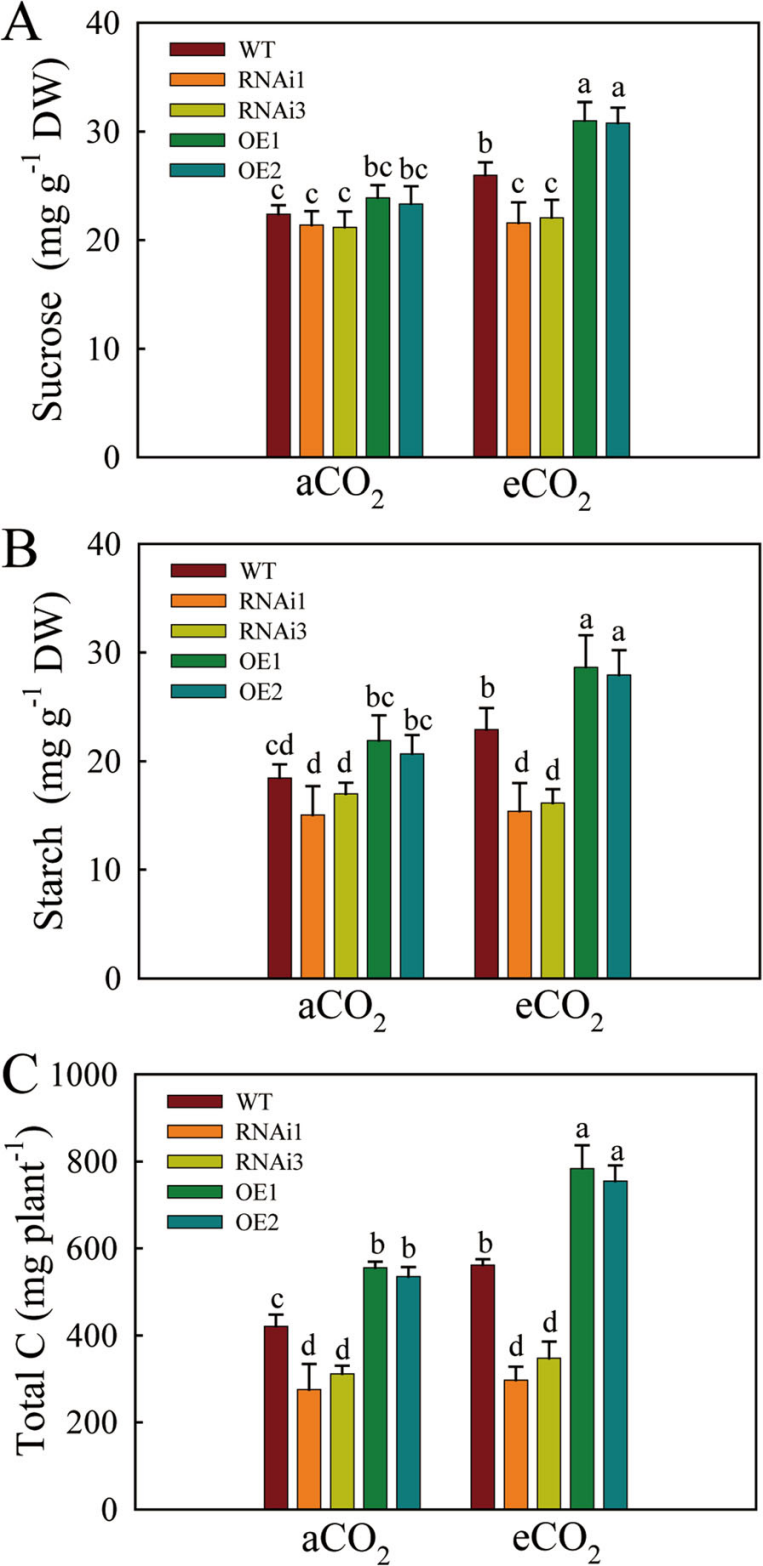
Effects of eCO_2_ on the contents of carbohydrates in WT, RNAi lines and OE lines of *qPE9-1*. (**A**) Sucrose (*n* = 5). (**B**) Starch (*n* = 5). (**C**) Total C (*n* = 5). All values are means ± SD; different letters indicate significant differences (*P* < 0.05)

In order to investigate the effect of *qPE9-1* transcript modulation in transgenic plants on photoassimilates, the sucrose, starch and total C content were determined in *qPE9-1* OE lines, RNAi lines and WT (Fig. 5). Compared to aCO_2_, the sucrose, starch concentration and total C content of the OE lines and WT were significantly higher in the presence of eCO_2_, whereas no significant difference was observed for RNAi lines. For sucrose, there was no significant difference between OE lines, RNAi lines and WT under aCO_2_, but the OE lines showed the highest sucrose concentration under eCO_2_ (Fig. 5 A). The starch concentration decreased by 8–18 % and 30–33 % of RNAi lines in comparison with WT under aCO_2_ and eCO_2_, respectively. Conversely, 12–18 % and 21–25 % higher starch concentrations were recorded in OE lines under aCO_2_ and eCO_2_, respectively (Fig. 5B). Compared with WT plants, the total C content was 27–32 % and 34–39 % higher in the OE lines under aCO_2_ and eCO_2_, while total C content in RNAi was 27–32 % and 34–39 % lower under aCO_2_ and eCO_2_ (Fig. 5 C).

### Effect of eCO_2_ on Rubisco content in rice leaves

**Fig. 6:**
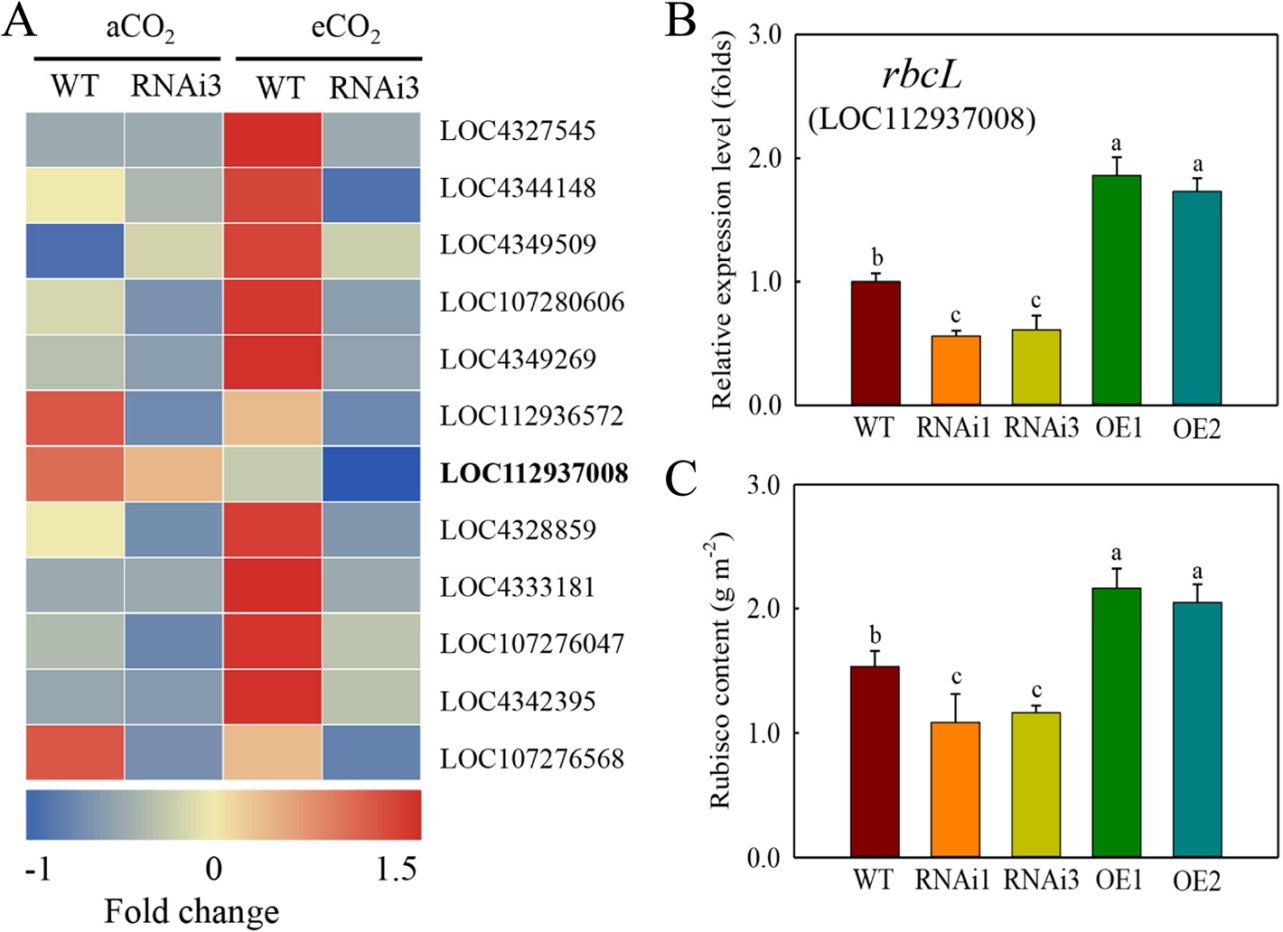
*qPE9-1* regulates Rubisco gene expression and its content in rice plants aCO_2_ or eCO_2_. Seedlings were grown in growth chambers under aCO_2_ (400 ppm) or eCO_2_ (800 ppm) for 4 weeks. (**A**)Heat map of the genes related to photosynthesis in leaves of WT and RNAi3 lines. The scale shows fold change, red indicates upregualtion and blue is downregulation. (**B**) Relative expression level of *rbcL* in leaves of WT and transgenic lines (*n* = 3). (**C**) The content of Rubisco in leaves of WT and transgenic lines (*n* = 5). All values are means ± SD; different letters indicate significant differences (*P* < 0.05)

To study how *qPE9-1* regulates leaf photosynthesis in response to eCO_2_, we used RNA-seq to determine the genes involved in photosynthesis. 3,095 DEGs (aCO_2_ vs. eCO_2_) were found in WT, comprising 1,993 up-regulated and 1,102 down-regulated genes (Fig. S3). In RNAi3, we found 3,020 DEGs (aCO_2_ vs. eCO_2_), out of which 1,854 genes were up-regulated and 1,166 genes were down-regulated (Fig. S3). Comparing RNAi3 with WT, 2,024 (1,078 up & 1,126 down) and 2,476 (1,153 up & 1,323 down) DEGs were found under aCO_2_ and eCO_2_ respectively (Fig. S3). 1,235 DEGs were shared between the “aCO_2_ WT vs. aCO_2_ RNAi3” and “eCO_2_ WT vs. eCO_2_ RNAi3”. GO term enrichment analysis was used to understand the functions of these DEGs. We mapped them to the three main categories, including biological process, cellular component and molecular function. According to biological process, the most abundant DEGs were involved in “cellular process” and “metabolic process”. In terms of cellular component, the genes were dominant in “cell part” and “cell”. “Catalytic activity” and “binding” two terms were enriched in molecular function (Fig. S5). According to GO term annotations, 128 genes were found to be involved in photosynthesis. Further, 12 photosynthesis-related genes were differently expressed in RNAi3 line relative to WT under eCO_2_ (Fig. 6 A, Table S3). For example, UDP-glycosyltransferase (LOC4327545) and magnesium-chelatase subunit (chloroplastic) (LOC4344148), which are involved in photosynthetic light reaction, were expressed higher in WT than in RNAi3 after exposure to eCO_2_. (Fig. 6 A, Table S3). 50 S ribosomal protein L2, chloroplastic (LOC107280606) and protein STRICTOSIDINE SYNTHASE-LIKE 5 (LOC4349269), which belongs to photosynthetic electron transport in photosystems II, showed higher gene expression in WT than in RNAi3 under eCO_2_. In addition, Rubisco large subunit (LOC112937008, *rbcL*) expression in the RNAi3 was lower than in WT under eCO_2_ (Fig. 6 A, Table S3).

qRT-PCR was also conducted to confirm the *rbcL* gene expression (in WT, OE and RNAi lines) under eCO_2_ (Fig. 6B). Compared to WT, the transcript level of *rbcL* in the leaves of OE lines was 73–86 % higher under eCO_2_. Whereas compared to WT, the expression level of *rbcL* was significantly decreased 39–44 % in RNAi lines under eCO_2_ (Fig. 6B). Compared to WT, the Rubisco content in the leaves of OE lines was about 33–41 % higher under eCO_2_, 24–29 % lower in RNAi lines under eCO_2_ (Fig. 6 C). The results showed that *qPE9-1* was involved in the regulation of photosynthesis under eCO_2_.

## Discussion

Numerous studies have been evaluated the responses of crops to eCO_2_ (Kim et al. 2003; Ainsworth 2008; Yang et al. 2007, 2020; Zhu et al. 2014; Xu et al. 2019; Becklin et al. 2017), but the role of *qPE9-1* in response to eCO_2_ remains unexplored. In the present study, we determined that G protein γ-subunit *qPE9-1* played a positive role in rice growth under eCO_2_ by regulating *A*_net_ and Rubisco content in leaves. G proteins are important signaling components in plants, which are involved in plant development and environmental responses (Urano and Jones 2014). Rice small GTPase, *OsRab6a*, which encodes monomeric G proteins related to the α-subunit of G proteins, played a positive role in rice growth under eCO_2_ conditions (Yang et al. 2020). So, based on this, we hypothesized that G protein γ-subunit *qPE9-1* might be involved in response to eCO_2_. In addition, according to Tanaka et al. (2016), there are three CCRE *cis*-elements (TGACGT, ACGTCA, and TGACGC) in response to eCO_2_ in the marine diatom *Phaeodactylum tricornutum*. Interestingly, the CCRE3 *cis*-element (TGACGC) was discovered in the promoter sequence of *qPE9-1* (Table S4), which indicates that *qPE9-1* might be involved eCO_2_ response. In addition, the expression level of *qPE9-1* was significantly increased under eCO_2_, compared to aCO_2_ (Fig. 1), while other G protein subunit genes (*RGA1*, *RGB1*, *RGG1*, *RGG2*, *GS3*, *GGC2*) were not induced by eCO_2_ (Fig. S1). The results suggest that *qPE9-1* may be important in response and adaptation to eCO_2_ in rice.

The *qPE9-1* OE and RNAi lines show the different growth phenotype to WT under aCO_2_ (Fig. 2 A), which suggests that the role of *qPE9-1* is in both aCO_2_ and eCO_2_. In the present study, the root biomass of RNAi lines was lower than WT; it is also possible that *qPE9-1* has different roles in shoot and root. For the role of *qPE9-1* in root, it is reported that G protein genes mutant can lead to unusual root elongation (Ullah et al. 2003; Chen et al. 2006). Then, rice *RGA1* is involved in root growth under the brassinosteroid response (Wang et al. 2006). On the other hand, sucrose, the major transport photosynthetic products (Sung et al. 1989), provides energy for root growth and development (Chiou and Bush 1998). In the present study, the *qPE9-1* RNAi lines showed lower root biomass than WT (Fig. 2), which is may be associated with the lower CO_2_ assimilation rate in RNAi lines (Fig. 4 A). The sucrose may be the connection between the regulation photosynthesis of *qPE9-1* and root biomass.

It is well known that eCO_2_ increases leaf photosynthesis (Widodo et al. 2003), induces stomatal closure (Uprety, 2002) and decreases transpiration (Baker and Allen 2005). The increased leaf photosynthesis under eCO_2_ will contribute to enhance shoot and root growth in C_3_ crop plants (Kim et al. 2003; Ainsworth 2008). In our study, the overexpression of *qPE9-1* resulted in significantly higher *A*_net_ than WT under eCO_2_ (Fig. 4 A), which suggests that *qPE9-1* may be involved in *A*_net_ regulation under eCO_2_. Our results are consistent with the observation in GTPase *Rab6a* overexpression rice plants, which suggests great potential of *Rab6a* in increasing rice *A*_net_ under eCO_2_ (Yang et al. 2020). Plant biomass is a complex trait and can be affected by many factors (Xing and Zhang 2010). Previous studies showed that growth did not correlate well with the rate of photosynthesis (Poorter and Remkes 1990; Honda et al. 2021). Additionally, *qPE9-1* was involved in regulating the genes related to “binding” using GO terms analysis under aCO_2_ (Fig. S4), which is associated with rice growth (Ya et al. 2014). So, the *qPE9-1* OE lines showed higher biomass probably by regulating genes related to “binding” under aCO_2_ conditions. According to Zhang et al. (2015), *qPE9-1* can negatively regulate stomatal conductance through modulating ABA signaling. In the present study, stomatal conductance and CO_2_ assimilation of RNAi lines was lower than WT under both aCO_2_ and eCO_2_ (Fig. 4). Thus, the ABA signaling may be involved in the difference of CO_2_ assimilation and stomatal conductance between WT and RNAi lines. Under aCO_2_, the carbohydrates concentrations of WT and OE lines showed no significantly increase relative to RNAi lines, which probably due to the “dilution effect” as a result of fast growth of WT and OE lines (Yang et al. 2002).

During photosynthetic light reaction, mainly adenosine triphosphate and nicotinamine adenine dinucleotide phosphate are produced through chloroplast photosynthetic electron transport and coupled photophosphorylation to support the light reaction (Zhang et al. 2008; De Souza et al. 2008) found that chlorophyll *a*-*b*-binding protein, ferredoxin-1, photosystem I (PSI) reaction centre subunit N and photosystem II (PSII) preotein K, which belong to the electron transport system, were all up-regulated in sugarcane leaves under eCO_2_. In the present study, the 11 DEGs (exception the *rbcL*) related photosynthetic genes showed significant changes in WT but not in RNAi3 under eCO_2_ (Fig. 6 A, Table S3), which suggests that *qPE9-1* is also involved in rice adaptation under elevated CO_2_ concentration by regulating rice photosynthetic light reaction. LOC112936572 and LOC107276568 were repressed in WT under eCO_2_, which probably because that some related photosynthetic genes show different expression under eCO_2_ (Fukayama et al. 2009, 2011). Rubisco is an essential protein in the Calvin-Benson cycle of plant photosynthesis (Kanno et al. 2017). As it is the first enzyme in CO_2_ fixation process, changes in photosynthesis rate is reflected in the Rubisco content and its gene expression level (Zhu et al. 2014). Furthermore, previous studies showed that the *rbcL* expression in rice and barely leaf was significantly decreased under eCO_2_ (Zhu et al. 2014; Torralbo et al. 2018). In the present study, the expression level of *rbcL* in WT and RNAi3 was down-regulated under eCO_2_ relative to under aCO_2_ (Fig. 6 A), and the *rbcL* expression level and Rubisco content were higher in WT than in RNAi3 (Fig. 6). The data suggest that *qPE9-1* may regulate rice response to eCO_2_ by regulating Rubisco content and its gene expression. Our results suggest that *qPE9-1* could regulate rice *A*_net_ by promting photosynthetic light reaction and Rubisco content under eCO_2_.

In conclusion, our results indicate that *qPE9-1* may be an important molecular regulator of photosynthesis and rice growth under eCO_2_ by moderating rice photosynthetic light reaction and Rubisco content. Taken together, the findings are pertinent to optimizing crop growth in future climate scenarios.

## Materials and methods

### Plant materials and growth conditions

Zhonghua 11 (ZH11) was used as wild-type (WT) in this study. Rice seeds were sterilized as described in Xu et al. (2019) for hydroponic experiments. After 7 d, rice seedlings were transplanted into 7-litre plastic containers, and grown in a plant growth chamber (Saifu DRX-680E-DG-CO_2_, Ningbo, China). The growth condition of the chamber was as follows: 300 µmol m^− 2^ s^− 1^ light intensity at shoot height, an approximately 60 % relative humidity, and a 14 h light (26 ℃)/10 h dark (22 ℃) photoperiod. Each experiment was randomized and involved three replicates of five plants each at two different carbon-dioxide concentrations of 400 ppm (aCO_2_) and 800 ppm (eCO_2_). The nutrient solution (pH 5.5) contained 1.25 mM NH_4_NO_3_, 0.3 mM K_2_SO_4_, 0.3 mM NaH_2_PO_4_, 1 mM CaCl_2_, 1 mM MgSO_4_, 9 µM MnCl_2_, 0.39 µM Na_2_MoO_4_, 20 µM H_3_BO_4_, 0.77 µM ZnSO_4_, 0.32 µM CuSO_4_, and 20 µM EDTA-Fe. Nutrient solution was exchanged every 3 days.

### Construction of ***qPE9-1***-transgenic rice plants

Generation of *qPE9-1* overexpression (OE) line was as described in Chen et al. (2016). Briefly, the open reading frame (ORF) sequence of *qPE9-1* was amplified using the primers listed in Table S1. The *qPE9-1* RNA-interference (RNAi) transgenic lines were generated in Zhou et al. (2009) and Zhang et al. (2015). The OE and RNAi transgenic plants were both generated in the *Oryza sativa* L. ssp. Japonica ZH11 rice background.

### Real-time quantitative PCR

To investigate the expression pattern of *qPE9-1*, rice samples were taken at different growth stages (Xu et al. 2019). The expression level of *qPE9-1* was determined in WT at the seedling and tillering stages grown in hydroponic system and three replications were made. In addition, to determine the effect of CO_2_ concentration on *qPE9-1* expression, 2 weeks old rice seedlings were grown at aCO_2_ (400 ppm) and eCO_2_ (800 ppm) in the plant growth chamber (Saifu DRX-680E-DG-CO_2_). Leaf samples were taken after transplantation at times: 0, 1, 3, 5, 7 and 14 d. TRIzol reagent was used for total RNA isolated (Invitrogen, Carlsbad, CA, USA). *qPE9-1* and *OsActin* transcripts were quantified in real-time quantitative RT-PCR using the primers listed in Tables S2 and the protocol of Weng et al. (2020).

### Gas exchange measurement

The LI-6400 instrument (LI-COR, Lincoln, NE, USA) was used for measuring gas exchange measurement in rice plants. The temperature of the leaf chamber was maintained at 25 ℃, and the photosynthetically active radiation (PPFD) was maintained at 1,000 µmol m^− 2^ s^− 1^. The CO_2_ concentration was adjusted to 400 or 800 ppm. The relative humidity in the leaf chamber was maintained at 50–60 %. Newly and fully expanded leaves were measured between 9:00–15:00 h daily.

### Plant carbohydrate and Carbon content measurement

Sucrose and starch content were measured as performed in Nakano et al. (1997). Dried plant material was ground to powder. Samples weighing around 0.1 g was extracted three times with 1 mL of 80 % (v/v) ethanol following by incubation in a boiling water bath for 5 min. Samples were then centrifuged at 12,000×g for 15 min. The combined supernatants were used for sucrose quantification. The 80 % ethanol-insolube fraction was used for starch quantification, as performed in Nakano et al. (1997). To analyze carbon content, plants were dried at 80 ℃ for 72 h and ground to powder. Then, 1 mg of powdered material per sample was loaded into small tin capsules and analyzed using a Flash 1112 Elemental Analyzer (Carbo Erba, Milan).

### RNA sequencing and data analysis

Rice seedlings of WT and RNAi3 were grown under aCO_2_ (400 ppm) and eCO_2_ (800 ppm) for 4 week in a growth chamber (DRX-680E-DG-CO_2_, Saifu, Ningbo, China). The youngest and fully expanded leaves were harvested for RNA sequencing (RNA-seq). Sequencing libraries were constructed using NEBNext Ultra (NEB, MA, USA) and sequenced using the BGISEQ-500 sequencer (BGI, Shenzhen, China). The assessment and removing low-quality reads were performed using the SOAPnuk (version 1.5.2). The high-quality reads were mapped to *Oryza*_*sativa*_Japonica_Group (IRGSP_1.0) transcripts using HISAT2 (version 2.0.4). Further procedures were performed according to Zhang et al. (2020). Differentially expressed genes (DEGs) were analyzed using DEGseq (version 1.18.0) package in Bioconductor in R software environment. The fragments per kilobase of transcript per million mapped reads (FPKMs) values were used to assess transcript abundance. Genes with a log_2_ fold change ≥ 1 or ≤ − 1, and adjusted p-value ≤ 0.05 were considered as DEGs. TBtools software was used for preparing heatmap visualizations (Chen et al. 2020). Briefly, the DEGs data were uploaded to the TBtools software, and the primary heatmap was generated and normalized. Then, the generated heatmap was exported for use.

### Rubisco content measurement

The Rubisco content of rice leaves was measured using SDS-PAGE method as described in Makino et al. (1985). Briefly, rice leaves were taken and stored in liquid nitrogen, immediately. 0.5 g sample was ground in buffer solution, which contained 50 mM Tris-HCl buffer (pH 8.0), 5 mM β-mercaptoethanol and 12.5 % (v/v) glycerol, and centrifuged for 15 min at 4 ℃ in 1,500×g. Then, supernatant solution was taken and mixed with dissolving buffer [2 % (w/v) SDS, 4 % (v/v) β-mercaptoethanol and 10 % (v/v) glycerol]. The mixed solution was treated at 100 ℃ for 1 min immediately. Next, the samples were loaded onto SDS-PAGE. Afterwards, the gel was washed with water for three times, and dyed in Coomassie blue staining solution (0.25 %) for 12 h. Then, the gels were decolorized until the background was colorless. The large subunits and relevant small subunits were put in a cuvette, which contained 2 mL formamide, and washed in a 50 ℃ water bath for 8 h. Then, the washed solution was determined at 595 nm and bovine serum albumin (BSA) was used as a standard.

### Statistical analysis

Data was analysed using SPSS 18.0 software (SPSS Inc., Chicago, IL, USA). The difference in the effect of CO_2_ concentration on *qPE9-1* transgene plants were assessed statistically by one-way ANOVA followed by Duncan test. Differences were considered significant at *P* < 0.05.

## Supplementary Information

**Additional file 1:**

**Additional file 2:**

**Additional file 3:**

## Data Availability

All data supporting the conclusions of this article are provided within the article and its additional files.
