# Supplementary material for: G protein γ subunit qPE9-1 is involved in rice adaptation under elevated CO2 concentration by regulating leaf photosynthesis
Source: Rice (N Y). 2021 Jul 15;14:67. doi: 10.1186/s12284-021-00507-7 (PMC8282829; doi:10.1186/s12284-021-00507-7)
Supplement: Supplementary file 1 — Additional file 1: [file 12284_2021_507_MOESM1_ESM.doc]

**Supporting information:**


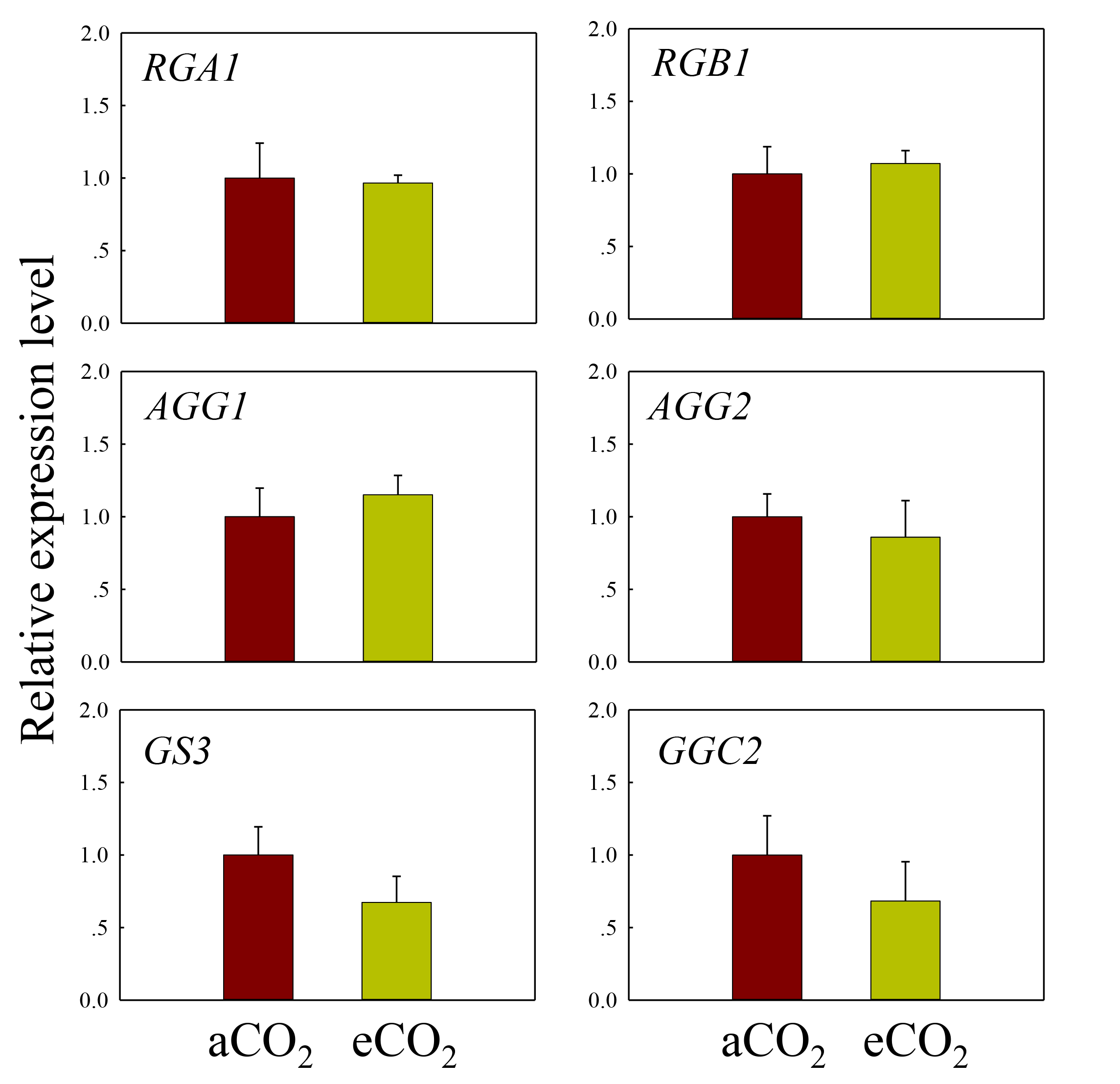


**Fig. S1** Transcript levels of different subunits of G proteins in WT under eCO_2_ concentration. WT seedlings were transferred to aCO_2_ and eCO_2_ conditions, then the leaves were harvested at 14 days for RNA extraction. *OsActin* was as an internal reference. Values are means±SD.


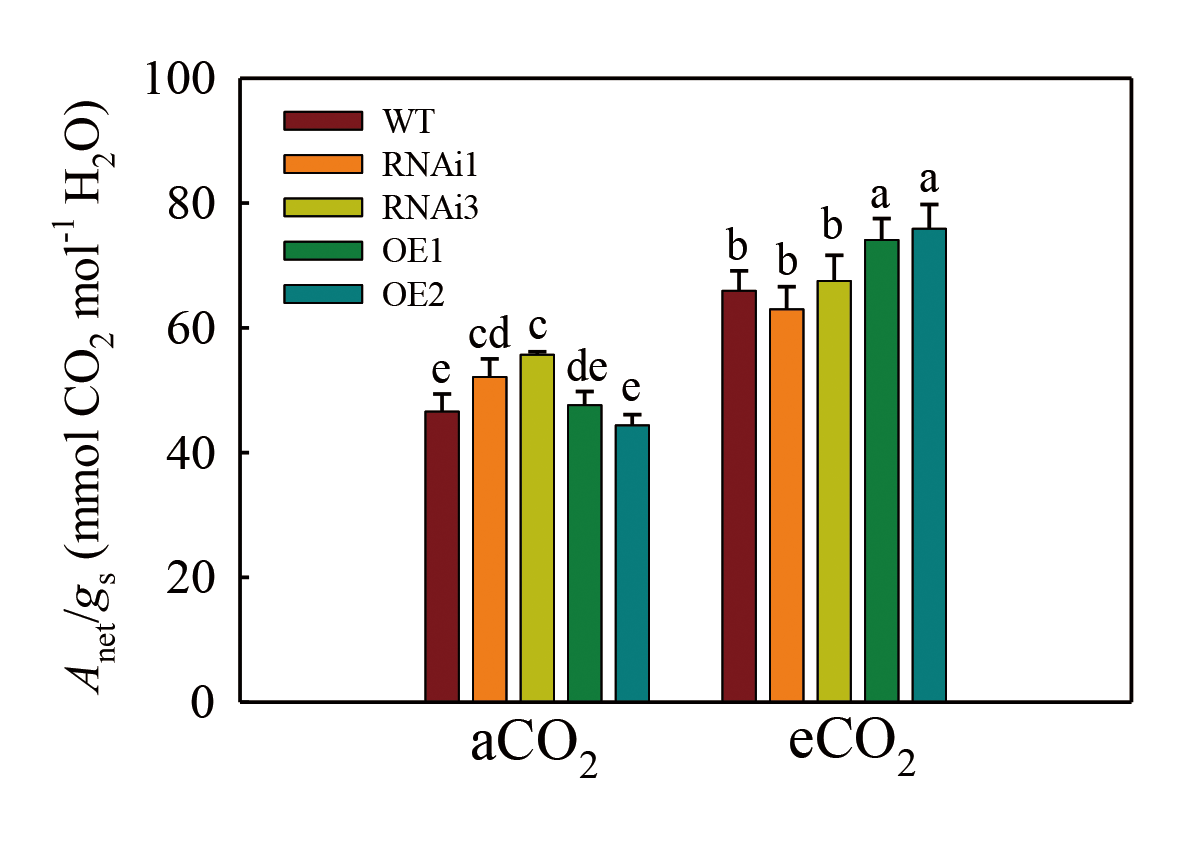


**Fig. S2** Effects of eCO_2_ on the ratio of CO_2_ assimilation (*A*_net_) and stomatal conductance (*g*_s_) in WT, RNAi lines and OE lines of *qPE9-1*. Values are means ± SD; different letters indicate significant differences (*P* < 0.05).


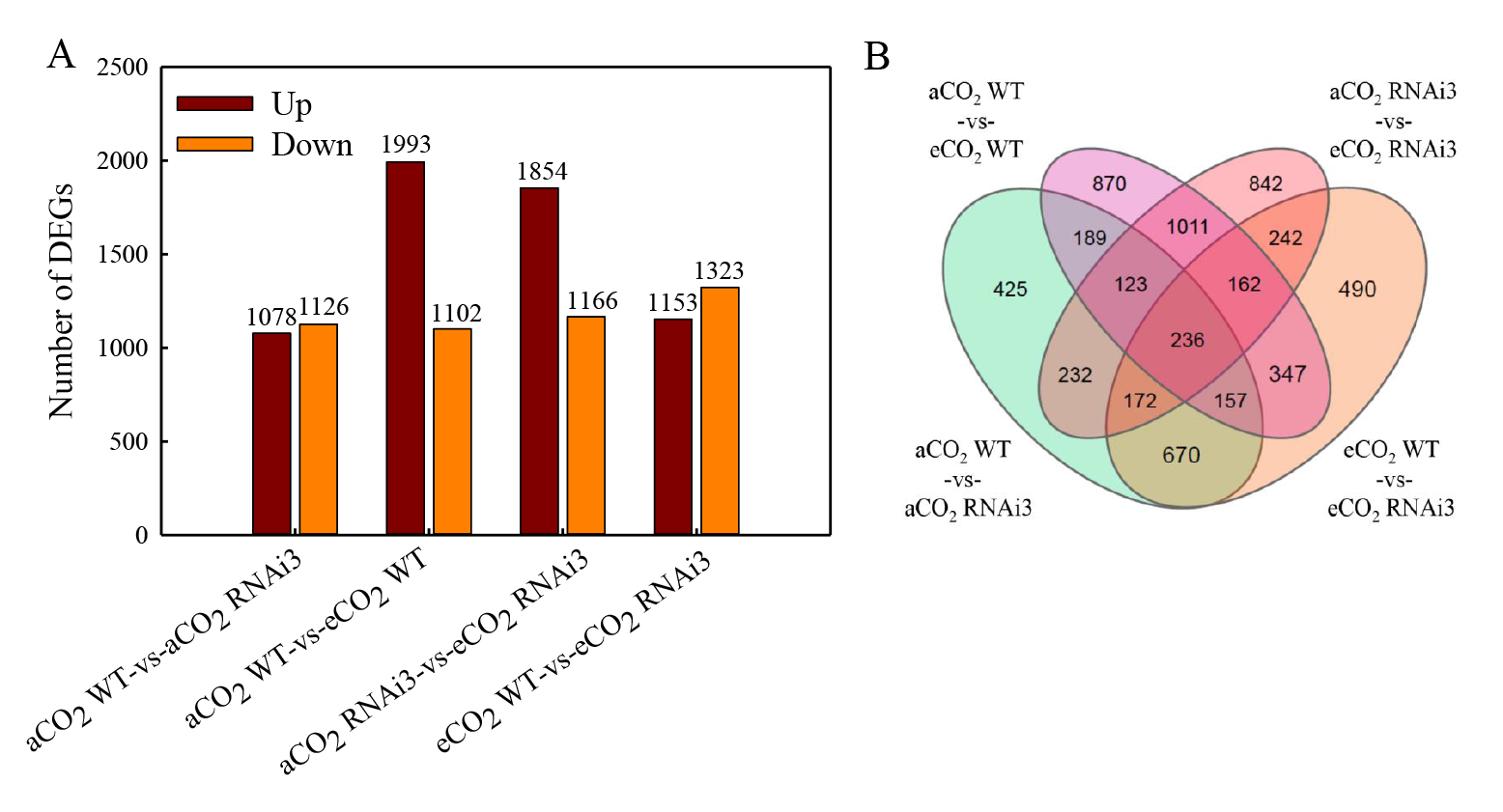


**Fig. S3** Comparison of DEGs in WT and RNAi3 after exposed to eCO_2_. (A) Numbers of up-regulated and down-regulated DEGs in WT and RNAi3 under aCO_2_ and eCO_2_ conditions. (B) Venn diagram illustrating the number of DEGs in WT and RNAi3 under aCO_2_ and eCO_2_ conditions.


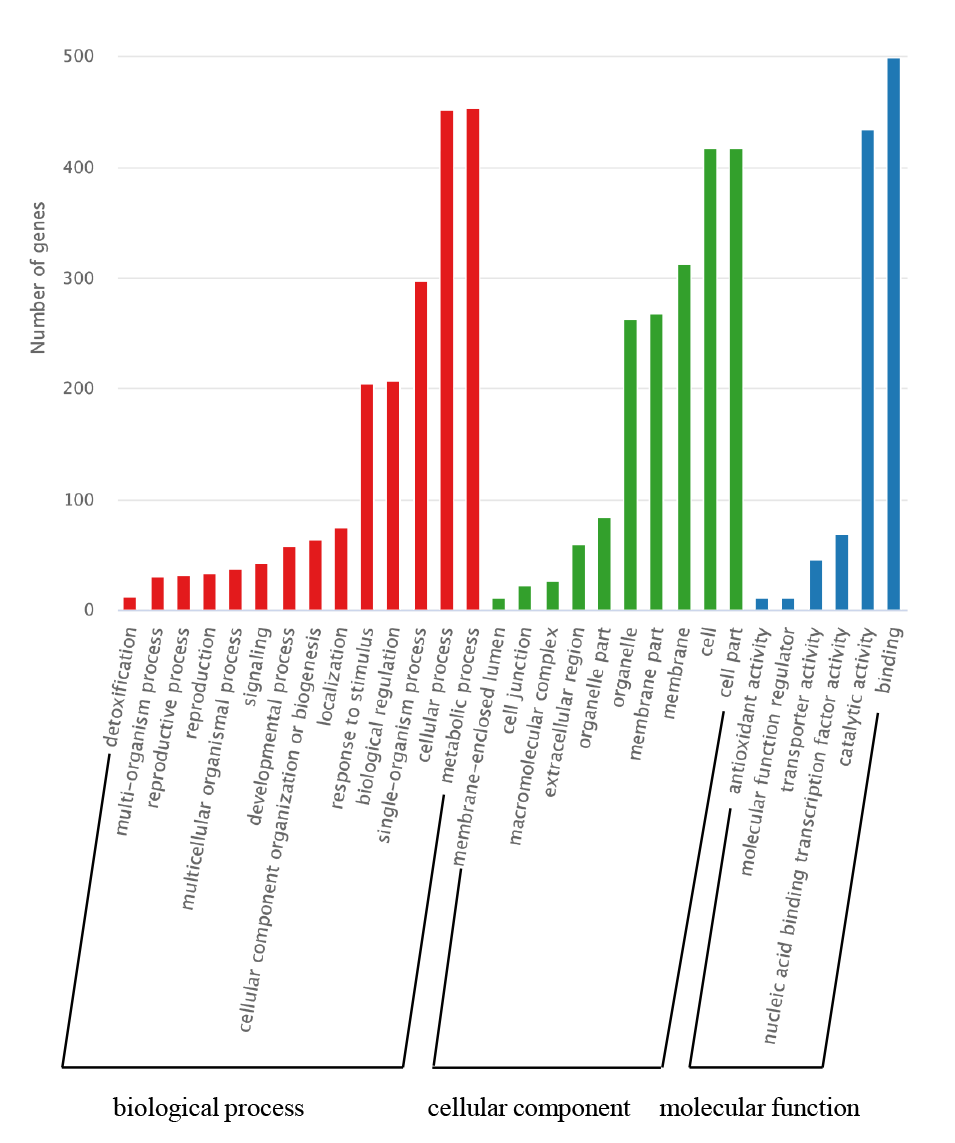


**Fig. S4** Significantly up-regulated genes were affected by *qPE9-1* under aCO_2_. The up-regulated genes in WT compared with RNAi3 under aCO_2_, with > 2-fold change. These up-regulated genes were functionally classified with GO terms and summarized in three main categories: molecular function, biological process and cellular component.


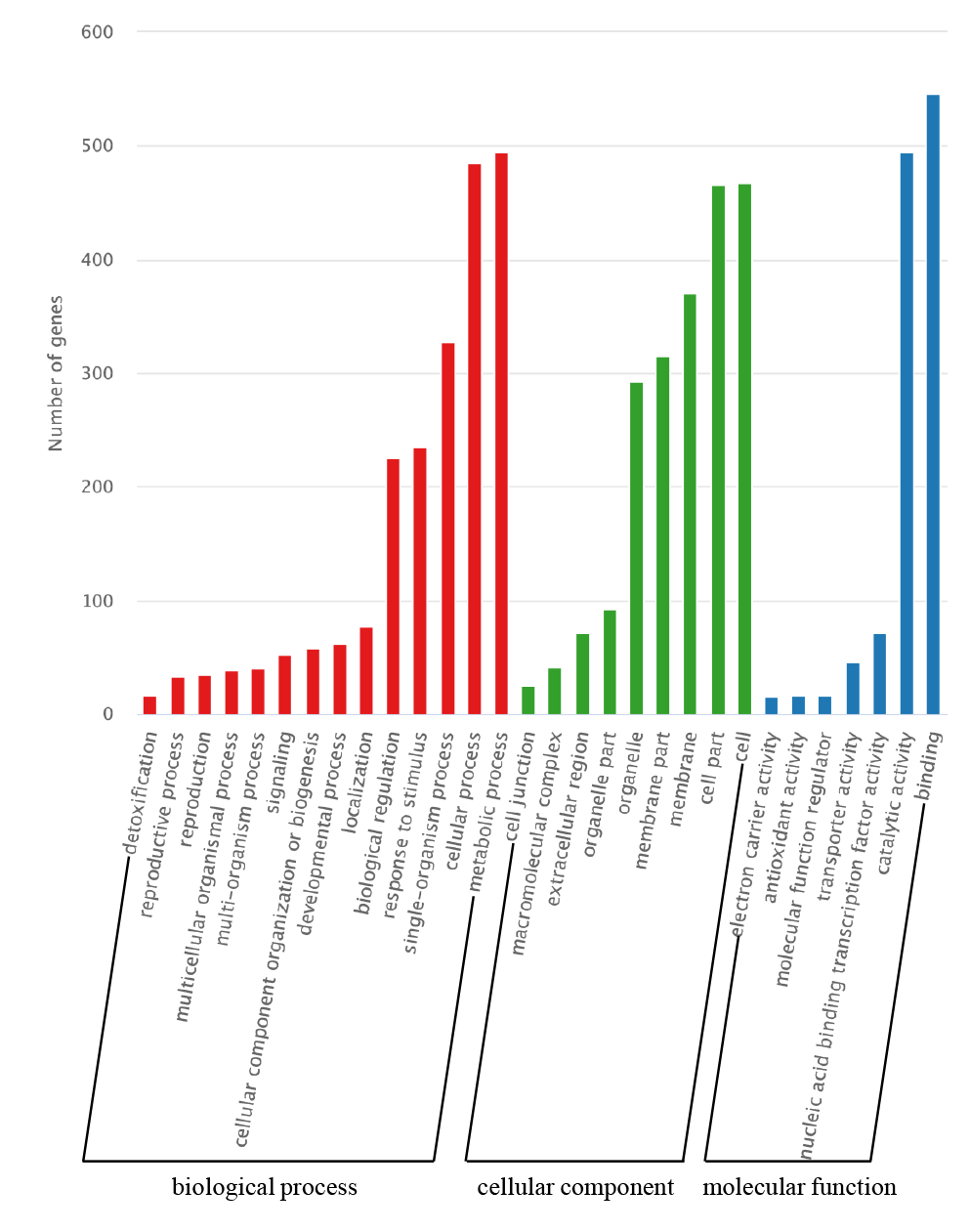


**Fig. S5** Number of differentially expressed genes (DEGs) associated with the most enriched GO terms in both "aCO_2_ WT vs aCO_2_ RNAi3" and "eCO_2_ WT vs eCO_2_ RNAi3".
