# Supplementary material for: G protein γ subunit qPE9-1 is involved in rice adaptation under elevated CO2 concentration by regulating leaf photosynthesis
Source: Rice (N Y). 2021 Jul 15;14:67. doi: 10.1186/s12284-021-00507-7 (PMC8282829; doi:10.1186/s12284-021-00507-7)
Supplement: Supplementary file 2 — Additional file 2: [file 12284_2021_507_MOESM2_ESM.doc]

**Supporting information:**

**Table S1** Primers used for construction of vector.

| Primer name | |  | Primer (5′-3′) |
| --- | --- | --- | --- |
| *qPE9-1*-OE | FP | | CAGTGGTCTCACAACATGGGGGAGGAGGCGGTGGT |
|  | RP | | CAGTGGTCTCATACATCAACATAAGCAACCACTGA |

**Table S2** Primers used for real-time quantitative PCR analysis.

| Gene name |  | Primer (5′-3′) |
| --- | --- | --- |
| *OsActin* | F | TGGCATCTCTCAGCACATTCC |
|  | R | TGCACAATGGATGGGCCAGA |
| *RGA1* | F | GGGAAACAGGAGGTTGAACA |
|  | R | GCGTCTCATGCTCT-CATCAA |
| *RGB1* | F | CTCACAAGATGGAAGGCTAA |
|  | R | CACCACAGGCAACAGATT |
| *RGG1* | F | CAAGAAGCTCGAGCAAGAGG |
|  | R | CGGACCTTCAAACCATCTGT |
| *RGG2* | F | TGCAGGATGAACTGAACGAG |
|  | R | GGATGCCCACCATTTGTTAC |
| *qPE9-1* | F | GGAGGAGGCGGTGGTGAT |
|  | R | CACCGAAAAAGACGGCAAG |
| *GS3* | F | CCGCGAGATCGGATTCC |
|  | R | CGTGGATCCCTTCGATTGA |
| *GGC2* | F | GTGCAACTGCTTGTTATGCC |
|  | R | GCTCGGTCTACAGCACGAT |
| *rbcL* | F | CTTGGCAGCATTCCGAGTAA |
|  | R | CAACGGGCCGATGTGATA |

**Table S4** A CCRE3 *cis*-element exists in the promoter of *qPE9-1*.

GTAGCAGCGGCGCGCTATCAGCTCTACCCGGAGTAGGCAGTAGCAGCAGTGGTGGTAGTAGTAGAAAGCGCGGCAGTGGAAGGGATAGAGGAGTGCCCACATTAATAACTCGTTTCAAATCGGACGGCGTGGCTACTCTAGTACACCCATTTCAACGGGACGTTCCACTCCACTCCACCGTAACTGCGCGCGTGGGGCGACCCCATCACGTGCCACGGTACGGGGGCGTCGCTGACCGTCTCTGGCCCACCGCAACCCTGCTCCCCCAATCCAAACCCCGCGCTGCACGTCTCGCTGGCACTGGGCATTTCGATCCATCGCGGTCGCGGCCGCGGTATCACGAC**TGACGC**GCGGGGCCGGCCCGGCACGCGAGACCGATGTAGACGTGTCACCGGGGAAGCTTGTCCGCTCGCGGTTGCCGCTGCGCGGGGGGGAGGGGAGGGCGTCGAGCCGTACGTCGTCGTCGCATACCTCGGTCGCGTCCCTGTCAAAGCCGGCCATCGCTGCCGGCTGCTCAATTTATTCCCTTGCTGTTTCATTTCGTACGTACTCCGCGCTCGGGATGCGGCCATAGCCATATCGCCATATATCTCGTGCAGGCACCGTCACGCTCGCTCGGCAACTGTACGTGCCGTCTCAGGACGCGGCAGTCAATGCGGAGTTGGTACAAACTTGCGATGAAAGGAGTGGTACTGTGGTACCACCACTGCCCTGTCTTTGCACACATCAGTCTTCCCGGAGTTTAGCTCAAGGAACGTGGGTGGGGGTGCTGTTTGTGTTTGTGTGTAGTTTTTGCCAATCCAAACAAATTGGCAAAGGGGGCTGGAAGAGTCCATGGTATTTCCCCTATTTGATTTGCAATTTGAACTGTGCAAAAAATAACGCGTGATTTCATCCCTGTACCACGGGACGAAGGATCGGCTTTGCATGGTCTGGGGAATCATGGCCCCATACCGGAGAAGTACCGGGCCCACGCAGCCATGATCCATCCCTCGAGACGGCCCGGCGACAGCGTATCCAACGTAGGCTCGGGCCCACGTCGCAGTGGAACAAAGCCCACAGACACAAAACCCCACCCCCCGCTCGCTGTTGAACTCTCTCTTCCCTCTCTCTCTTTCTCTCTCCAAACCCCACGCACGCCGCGTCGCCGCCTCCTCCTCTCCATCTCCGCTGCTATTATTGCCCGCGCAGACGCAGGCCACCATCCTTCCTCTCGCTCACGCTCGCTGCTATATGGGGGTCCTCCTCATCGCATCGCATCGCATCACCTCGCACGGGCGCGCGCGCCGTGCCGTGCCGCTAGCTCGATCCGCCTCGTACGCCAGCTCGCTCGCTCGCTCCCCCACCCCGCTGCTGCACGGCTGCGCCCGCGCTGTCCCCTGTCCCCCCGCTCGCCGCGGCGATTTATACCCACCACGCCCCCTGCTGCTGCTATAATGCCCATGAGTGAAGGCGGCGAGGGGTGGTTCTGAGTTGGCCGTTGGCGTGCTGCGTGTGGAGATG
